# Supplementary material for: Practice of preventive measures and vaccine hesitance for COVID 19 among households in The Gambia, 2021: Study protocol
Source: PLoS One. 2022 Aug 30;17(8):e0270304. doi: 10.1371/journal.pone.0270304 (PMC9426907; doi:10.1371/journal.pone.0270304)
Supplement: S1 File — (DOCX) [file pone.0270304.s001.docx]

**PARTICIPANT INFORMATION SHEET and Consent form**

| Version | 2 | Date | 26-08-2021 |
| --- | --- | --- | --- |

Study Title: Practice of preventive measures and vaccine hesitance for COVID 19 among Households in The Gambia, 2021

Sponsor & Funder: WHO & MoH

## What is informed consent?

You are invited to take part in a research study. Participating in a research study is not the same as getting regular medical care. The purpose of normal medical care is to improve one’s health. The purpose of a research study is to gather information that may be useful in the future for the whole population. It is your decision to take part and you can stop at any time without giving any reason.

Before you decide you need to understand why the study is being done and what will happen in it. Please take time to read the following information or get the information explained to you in your language. Listen carefully. You can ask questions if there is anything that you do not understand. Ask for it to be explained until you are satisfied. You may also wish to speak your spouse, family members or others before deciding to take part in the study.

If you decide to join the study, you will need to sign or thumbprint a consent form saying you agree to be in the study. You will receive a copy of the consent form.

## Why is this study being done?

We will conduct this study to find out an accurate picture of what the general population knows about the pandemic together with their attitudes and practices towards prevention and control measures for this disease. This will help to guide the design and implementation of behaviour change communication strategies which will eventually lead to positive behaviour and the control of this Corona virus disease (COVID-19).

.

## What does this study involve?

This study involves collecting information from participants through the administration of a questionnaire with regard to their knowledge, attitudes and practices of COVID-19 in their communities or households in relationship to COVID 19 vaccination.

## What harm or discomfort can you expect in the study?

There is no expected harm or discomfort for participants in this study. However social distancing will be observe by all the data collectors to protect them from this virus as well as the wearing of the face mask and the use of hand sanitizer when water and soap is not available. The interviewee will have to answer the questionnaire for 60 minutes.

## What benefits can you expect in the study?

There is no direct benefit to participants from this study, but the data obtained from participants will be analysed to provide information to policy makers and other actors to plan and implement relevant interventions to control and prevent COVID-19 infection in this country.

## Will you be compensated for participating in the study?

You will not get paid by the study, and the study teams will not provide any financial benefit to participants.

## What happens if you refuse to participate in the study or change your mind later?

You are free to join the study or not and you are free to stop being in the study any time without giving a reason. You will still get the normal medical care at any public health facility.

If you do not want to continue in the study we will use only the information already collected from you.

## How will personal records remain confidential and who will have access to it?

All information that is collected about you in the study will be kept strictly confidential. Your personal information without your name will only be seen by the study team members, the sponsor and if necessary the Ethics Committee and Government authorities.

## Who should you contact if you have questions?

If you have any questions or are worried you can call **Mr. Sainey Sanneh on +220 2122669 / 3247910, Dr. Sharmila LAREEF-JAH on / +220 3300200 or Mr. Buba Darboe on +220 7059325.** Please feel free to ask any question you might have about the study.

## Who has reviewed this study?

This study has been checked by scientists at the Medical Research Council and by the Gambia Government/MRC Joint Ethics Committee. The Ethics Committee protects your rights and wellbeing, and has given permission for it to take place.

**Consent Form**

Participant Identification Number: |__|__|__|__|__|__|__|__|__|__|__|__|

(Printed name of participant)

I have read the written information **OR**

I have had the information explained to me by study personnel in a language that I understand,

and I

- confirm that my choice to participate is entirely voluntarily,
- confirm that I have had the opportunity to ask questions about this study and I am happy with the answers that have been provided,
- understand that I allow access to the information about me by the persons described in the information sheet,
- had enough time to think about whether I want to take part in this study,
- agree to take part in this study.

| Participant’s signature/ thumbprint* |  |  |  |  |
| --- | --- | --- | --- | --- |
|  |  |  | Date (dd/mmm/yyyy) Time (24hr) | |
|  |  |  |  | |
| Signature / thumbprint of witness* |  | | | |
| Signature / thumbprint of person obtaining consent |  | | | |
| **I attest that I have explained the study information accurately in** ____________________**__ to, and was understood to the best of my knowledge by, the participant. He/she has freely given consent to participate ***in the presence of the above signed witness (where applicable).** | | | | |
| Signature of person obtaining consent |  |  |  | |
|  |  |  | Date (dd/mmm/yyyy) Time (24hr) | |
| ** Only required if the participant is unable to read or write.* | | | | |
