# Supplementary material for: Practice of preventive measures and vaccine hesitance for COVID 19 among households in The Gambia, 2021: Study protocol
Source: PLoS One. 2022 Aug 30;17(8):e0270304. doi: 10.1371/journal.pone.0270304 (PMC9426907; doi:10.1371/journal.pone.0270304)
Supplement: S2 File — (DOCX) [file pone.0270304.s002.docx]

Title:Practice of preventive measures and vaccine hesitance for COVID 19 among Households in The Gambia, 2021

| *READ: Hello I am working on a study for the Ministry of Health Supported by WHO. The purpose of the study is to collect information that will help to strengthen our response to COVID-19. I would like to ask you some questions about what you know about the disease. Your answers are confidential and cannot be linked back to you. Your participation is completely voluntary, and you may decline to answer any specific question or completely refuse to participate. The interview should take about 60 minutes of your time and you will not be contacted in the future. We would greatly appreciate your help in responding to these questions.* | | | |  |  |  |
| --- | --- | --- | --- | --- | --- | --- |
| *Are you willing to answer these questions?*  **IF NO, STOP INTERVIEW* | | | YES 1  NO 2 |  |  |  |
| **#** | **QUESTIONS** | | **CODE** |  |  |  |
| Q1 | Interview Number | |  |  |  |  |
| Q2 | Interviewer initials | |  |  |  |  |
| Q3 | Location | | Region:_________________________________  District of Residence: ­­­­­____________________  Village__________________________________ |  |  |  |
| Q4 | Date of Interview | | _________________  DD / MM / YY |  |  |  |
| **DEMOGRAPHICS** | | | |  |  |  |
| Q5 | Gender | | MALE 1  FEMALE 2  OTHER 3 |  |  |  |
| Q6 | a. How old were you on your last birthday?  b. In what year were you born? | | AGE IN YEARS: __________    YEAR OF BIRTH: __________  YYYY |  |  |  |
| Q7 | What is your current marital status? | | SINGLE 1  MARRIED 2  CO-HABITING 3  DIVORCED 4  WIDOWED 5  SEPARATED 6  NOT STATED 7  OTHER (SPECIFY) 8 |  |  |  |
| Q8 | Religion | | ISLAM 1  CHRISTIANITY 2  OTHER 3 |  |  |  |
| Q9 | What occupation best describes the main type of work you currently do for a living? | | UNEMPLOYED 1  BUSINESS (except petty trader) 2  PETTY TRADER 3  PUBLIC TRANSPORTATION DRIVER 4  HEALTH PERSONNEL 5  OTHER GOVT EMPLOYEES 6  STUDENT 7  PHYSICAL LABOUR 8  OTHER 9 |  |  |  |
| Q10 | What is your highest level of education completed? | | NO FORMAL EDUCATION 1  PRIMARY SCHOOL (LOWER BASIC) 2  BASIC CYCLE 3  SECONDARY SCHOOL (UPPER BASIC) 4  SENIOR SECONDARY 5  DIPLOMA 6  BACHELOR’S DEGREE 7  MASTERS DEGREE OR HIGHER 8  OTHER (SPECIFY) 9 |  |  |  |
| **KNOWLEDGE** | | | | | | |
| Q11 | Have you heard of COVID-19?  (If No, please inform your supervisor of this location) | | | YES 1  NO 2  I DON’T KNOW/NOT SURE 3  NO RESPONSE 4 | | |
| Q12 | What is your main source of information?   \| Information source \| YES (1) \| NO (2) \| I DON’T KNOW/NOT SURE (3) \| \| --- \| --- \| --- \| --- \| \| TV \|  \|  \|  \| \| Radio \|  \|  \|  \| \| WhatsApp \|  \|  \|  \| \| Friends \|  \|  \|  \| \| Family members \|  \|  \|  \| \| Health care worker \|  \|  \|  \| \| Religious leaders \|  \|  \|  \| \| Community leaders \|  \|  \|  \| \| Other community mobilisers \|  \|  \|  \| \| Community members \|  \|  \|  \| | | | | | |
| Q13 | Which source do you trust the most to receive information related to coronavirus?   \| Information source \| YES (1) \| NO (2) \| I DON’T KNOW/NOT SURE (3) \| \| --- \| --- \| --- \| --- \| \| TV \|  \|  \|  \| \| Radio \|  \|  \|  \| \| WhatsApp \|  \|  \|  \| \| Friends \|  \|  \|  \| \| Family members \|  \|  \|  \| \| Health care worker \|  \|  \|  \| \| Religious leaders \|  \|  \|  \| \| Community leaders \|  \|  \|  \| \| Community mobilisers \|  \|  \|  \| \| Community members \|  \|  \|  \| | | | | | |
| Q14 | Are you aware that The Gambia has cases of COVID-19? | | | YES 1 NO 2  I DON’T KNOW/NOT SURE 3  NO RESPONSE 4 | | |
| Q15 | What causes COVID-19?   \| CAUSES \| YES (1) \| NO (2) \| I DON’T KNOW/NOT SURE (3) \| \| --- \| --- \| --- \| --- \| \| Virus \|  \|  \|  \| \| Eating bats \|  \|  \|  \| \| Black magic \|  \|  \|  \| \| Curse \|  \|  \|  \| \| God/Higher power \|  \|  \|  \| | | | | | |
| Q16 | Which of these ways is COVID-19 transmitted?   \| WAYS OF TRANSMISSION \| YES (1) \| NO (2) \| I DON’T KNOW/NOT SURE (3) \| \| --- \| --- \| --- \| --- \| \| Airborne \|  \|  \|  \| \| Through respiratory droplets from infected person \|  \|  \|  \| \| Direct contact with an infected person \|  \|  \|  \| \| Mosquito bites \|  \|  \|  \| \| Touching Contaminated objects/surfaces \|  \|  \|  \| \| Drinking unclean water \|  \|  \|  \| | | | | | |
| Q17 | \| SYMPTOMS \| YES (1) \| NO (2) \| I DON’T KNOW/NOT SURE (3) \| \| --- \| --- \| --- \| --- \| \| Fever \|  \|  \|  \| \| Fatigue \|  \|  \|  \| \| Dry cough \|  \|  \|  \| \| Body pains \|  \|  \|  \| \| Loss smell \|  \|  \|  \| \| Loss taste \|  \|  \|  \| \| Shortness of breath \|  \|  \|  \|   The following are the main signs and symptoms of COVID-19 | | | | | |
| Q18 | Do you know that there is a toll-free number (1025) to report your symptoms | | | YES 1 NO 2  I DON’T KNOW/NOT SURE 3  NO RESPONSE 4 | | |
| Q19 | Do you know currently there is no effective cure for COVID-19? | | | YES 1 NO 2  I DON’T KNOW/NOT SURE 3  NO RESPONSE 4 | | |
| Q20 | Do you belief that taking COVID 19 vaccination could protect you from developing severe complication associated with COVID infections | | | YES 1 NO 2  I DON’T KNOW/NOT SURE 3  NO RESPONSE | | |
| Q21 | Do you belief that COVID 19 vaccination could cause infertility? | | | YES 1  NO 2  I DON’T KNOW/NOT SURE 3  NO RESPONSE | | |
| Q22 | Do you believe that the hot climate will stop the transmission of COVID-19 in The Gambia? | | | YES 1 NO 2  I DON’T KNOW/NOT SURE 3  NO RESPONSE 4 | | |
| Q23 | Do you think eating garlic is a way of preventing/ cure COVID-19? | | | YES 1 NO 2  I DON’T KNOW/NOT SURE 3  NO RESPONSE 4 | | |
| Q24 | Do you think eating ginger /drinking ginger juice is a way of preventing/cure COVID-19? | | | YES 1 NO 2  I DON’T KNOW/NOT SURE 3  NO RESPONSE 4 | | |
| Q25 | Do you think eating Bitter kola seeds /drinking its juice is a way of preventing COVID-19 | | | YES 1  NO 2  I DON’T KNOW/NOT SURE 3  NO RESPONSE 4 | | |
| Q26 | Do you think traditional medicine could cure COVILD 19? | | | YES 1  NO 2  I DON’T KNOW/NOT SURE 3  NO RESPONSE 4 | | |
| Q27 | Do you think staying 1 meter or more from other is a way to prevent COVID-19? | | | YES 1 NO 2  I DON’T KNOW/NOT SURE 3  NO RESPONSE 4 | | |
| Q28 | Can one prevent oneself from getting COVID-19 by frequent hand washing? | | | YES 1 NO 2  I DON’T KNOW/NOT SURE 3  NO RESPONSE 4 | | |
| Q29 | Can putting on face mask prevent oneself and others from getting COVID-19 | | | YES 1 NO 2  I DON’T KNOW/NOT SURE 3  NO RESPONSE 4 | | |
| Q30 | Is ‘not touching the face, eyes, nose and mouth’ is way of preventing COVID-19 infection? | | | YES 1 NO 2  I DON’T KNOW/NOT SURE 3  NO RESPONSE 4 | | |
| Q31 | Is avoiding crowded places a way of preventing COVID-19? | | | YES 1 NO 2  I DON’T KNOW/NOT SURE 3  NO RESPONSE 4 | | |
| Q32 | Is restricting non-essential public transport a way of preventing COVID-19? | | | YES 1 NO 2  I DON’T KNOW/NOT SURE 3  NO RESPONSE 4 | | |
| Q33 | Do you think isolating infected people is a way of preventing the spread of COIVD-19? | | | YES 1 NO 2  I DON’T KNOW/NOT SURE 3  NO RESPONSE 4 | | |
| Q34 | Who do you think is at highest risk to get the coronavirus?   \| Group \| YES (1) \| NO (2) \| I DON’T KNOW/NOT SURE (3) \| \| --- \| --- \| --- \| --- \| \| Children \|  \|  \|  \| \| Youth \|  \|  \|  \| \| Adults \|  \|  \|  \| \| Elderly people \|  \|  \|  \| \| People with diabete,HIV and other health complications \|  \|  \|  \| \| Health care workers \|  \|  \|  \| | | | | | |
| **ATTITUDE** | | | | | | |
| Q35 | Do you trust the COVID 19 information from the National Authorities such as Ministry of Health? | | | YES 1 NO 2  I DON’T KNOW/NOT SURE 3  NO RESPONSE 4 | | |
| Q36 | What level of risk do you think  you have in getting COVID-19? | | | NO RISK 1  LOW RISK 2  MEDIUM RISK 3  HIGH RISK 4  I DON’T KNOW/NOT SURE 5  NO RESPONSE 6 | | |
| Q37 | Do you consider it important to take actions to prevent the spread of COVID-19 in The Gambia? | | | YES 1 NO 2  I DON’T KNOW/NOT SURE 3  NO RESPONSE 4 | | |
| Q38 | Do you consider it important to take actions to mandatory vaccination all eligible Gambian population to the spread of COVID-19 in The Gambia? | | | YES 1 NO 2  I DON’T KNOW/NOT SURE 3  NO RESPONSE 4 | | |
| Q39 | Will you recommend your family to be vaccinated against COVID 19? | | | YES 1 NO 2  I DON’T KNOW/NOT SURE 3  NO RESPONSE 4 | | |
| **PRACTICES** | | | | | | |
| Q40 | Since the COVID-19 outbreak, have you gone to any crowded place? | | | YES 1  NO 2  I DON’T KNOW/NOT SURE 3  NO RESPONSE 4 | | |
| Q41 | In recent days, have you been washing your hands with soap and water more often | | | YES 1  NO 2  I DON’T KNOW/NOT SURE 3  NO RESPONSE 4 | | |
| Q42 | In recent days, have you been washing your hands with soap and water more often | | | YES 1  NO 2  I DON’T KNOW/NOT SURE 3  NO RESPONSE 4 | | |
| Q43 | Have you being vaccinated against COVID 19 | | | YES 1  NO 2  I DON’T KNOW/NOT SURE 3  NO RESPONSE 4 | | |
| Q44 | If no, what could have being the reasons | | |  | | |
| Q45 | Have you being voluntarily tested for COVID 19? | | | YES 1  NO 2  I DON’T KNOW/NOT SURE 3  NO RESPONSE 4 | | |
| Q46 | If no, what could have being the reasons | | |  | | |
| Q47 | If you or your family member developed symptoms of COVID-19, what would you do?   \|  \| YES (1) \| NO (2) \| I DON’T KNOW/NOT SURE (3) \| \| --- \| --- \| --- \| --- \| \| Go to a pharmacy \|  \|  \|  \| \| Go to a traditional healer \|  \|  \|  \| \| Go to the nearest hospital \|  \|  \|  \| \| Call 1025 and report \|  \|  \|  \| \| Will wait for it to get better on its own \|  \|  \|  \| | | | | | |
| Q48 | In recent days, have you maintained a distance of at least 1m between yourself and others when out in public? | | | YES 1  NO 2  I DON’T KNOW/NOT SURE 3  NO RESPONSE 4 | | |
