# Supplementary material for: Practice of preventive measures and vaccine hesitance for COVID 19 among households in The Gambia, 2021: Study protocol
Source: PLoS One. 2022 Aug 30;17(8):e0270304. doi: 10.1371/journal.pone.0270304 (PMC9426907; doi:10.1371/journal.pone.0270304)
Supplement: Attachment — Submitted filename: reviewers comments.pdf [file pone.0270304.s004.pdf]

PLOS ONE

Knowledge, Attitude and Practices of COVID19 and associated vaccine hesitance among Households in the Gambia, 2021.Study protocol.

--Manuscript Draft--

**Manuscript Number:**	PONE-D-21-36320
**Article Type:**	Study Protocol
**Full Title:**	Knowledge, Attitude and Practices of COVID19 and associated vaccine hesitance among Households in the Gambia, 2021.Study protocol.
**Short Title:**	Knowledge, Attitude and Practices of COVID19 among households in the Gambia.
**Corresponding Author:**	Bakary Sanneh, MSc.<br>National Public Health Laboratories<br>Banjul, Gambia GAMBIA
**Keywords:**	knowledge; attitude; practices; Vaccination hesitance; Outbreak; Covid19; Households; The Gambia
**Abstract:**	The first imported confirmed case of COVID-19 was reported in The Gambia on 16 th of March 2020 leading to the implementation of relevant public health interventions to prevent further importation and spread of the virus. However, by 8th November, 2021 the country had registered cumulatively 9.980 COVID-19 confirmed infection and 341 deaths. The country has develop and implemented Risk Communication and Community Engagement (RCCE) Action Plan since the declaration by WHO that COVID-19 outbreak as global public health threat and subsequent proclamation of the outbreak as a pandemic. Despite the sensitization, some Gambians are in denial and or misinformed of the existence of infection in the country. It is also evident that social distancing and other restrictions have not been adequately implemented by the citizenry. Only less 14% of the Gambian population have been vaccinated despite gross vaccine hesitance and disbelief. There is urgent need to investigate the knowledge, attitude and practices among the Gambians about preventive practices to control COVID-19. The proposed study will enrol 1200 households from seven Local Government Areas (LGAs). The findings of this study will inform the messaging and health promotion activities to better inform the population to comply and practices preventive approach especially the use of mask, getting vaccinated in order to reduce the negative impact of COVID-19 outbreak in The Gambia and thus as a prospective to returning to new normal life.
**Order of Authors:**	Bakary Sanneh, MSc.
	Sainey Sanneh, MSc
	Sharmila Lareef-Jah, MSc
	Bakary Sanneh, MSc
	Lamin Dibba, MSc
	Lamin F. Manjang, BSc
	Yorro Bah, MSc
	Jalimory Suso, MSc
	Phebian Sangia, MSc
	Bakary Sanneh, MSc
	Sana M Sambou, MSc
**Additional Information:**	
**Question**	**Response**
**Financial Disclosure**	Will be Funded by World Health Country Office,The Gambia.Funding number of AFGMB2018552

Powered by Editorial Manager® and ProduXion Manager® from Aries Systems Corporation

Enter a financial disclosure statement that describes the sources of funding for the work included in this submission. Review the submission guidelines for detailed requirements. View published research articles from *PLOS ONE* for specific examples.<br><br>This statement is required for submission and **will appear in the published article** if the submission is accepted. Please make sure it is accurate.<br><br>**Unfunded studies**<br>Enter: *The author(s) received no specific funding for this work.*<br><br>**Funded studies**<br>Enter a statement with the following details:<br>• Initials of the authors who received each award<br>• Grant numbers awarded to each author<br>• The full name of each funder<br>• URL of each funder website<br>• Have or will the sponsors or funders play any role in the study design, data collection (when applicable) and analysis, decision to publish, or preparation of the manuscript?<br>• **NO** - Include this sentence at the end of your statement: *The funders had and will not have a role in study design, data collection and analysis, decision to publish, or preparation of the manuscript.*<br>• **YES** - Specify the role(s) played.<br><br>* typeset	
**Competing Interests**<br><br>Use the instructions below to enter a competing interest statement for this submission. On behalf of all authors, disclose any competing interests that could be perceived to bias this work—acknowledging all financial support and any other relevant financial or non-financial competing interests.<br><br>This statement is required for submission and **will appear in the published article** if the submission is accepted. Please make	NO authors have competing interests

sure it is accurate and that any funding sources listed in your Funding Information later in the submission form are also declared in your Financial Disclosure statement.<br><br>View published research articles from *PLOS ONE* for specific examples.<br><br>**NO authors have competing interests**<br><br>Enter: *The authors have declared that no competing interests exist.*<br><br>**Authors with competing interests**<br><br>Enter competing interest details beginning with this statement:<br><br>*I have read the journal's policy and the authors of this manuscript have the following competing interests: [insert competing interests here]*<br><br>* typeset	
**Ethics Statement**<br><br>Enter an ethics statement for this submission. This statement is required if the study involved:<br><br>• Human participants<br>• Human specimens or tissue<br>• Vertebrate animals or cephalopods<br>• Vertebrate embryos or tissues<br>• Field research<br><br>Write "N/A" if the submission does not require an ethics statement.<br><br>General guidance is provided below. Consult the submission guidelines for detailed instructions. **Make sure that all information entered here is included in the Methods section of the manuscript.**	The study protocol was approved by the Gambia Government and MRCG Joint Ethic Committee. The approval number is :<br>Project ID/ethics ref: 22699

**Format for specific study types**<br><br>**Human Subject Research (involving human participants and/or tissue)**<br>• Give the name of the institutional review board or ethics committee that approved the study<br>• Include the approval number and/or a statement indicating approval of this research<br>• Indicate the form of consent obtained (written/oral) or the reason that consent was not obtained (e.g. the data were analyzed anonymously)<br><br>**Animal Research (involving vertebrate animals, embryos or tissues)**<br>• Provide the name of the Institutional Animal Care and Use Committee (IACUC) or other relevant ethics board that reviewed the study protocol, and indicate whether they approved this research or granted a formal waiver of ethical approval<br>• Include an approval number if one was obtained<br>• If the study involved *non-human primates*, add *additional details* about animal welfare and steps taken to ameliorate suffering<br>• If anesthesia, euthanasia, or any kind of animal sacrifice is part of the study, include briefly which substances and/or methods were applied<br><br>**Field Research**<br><br>Include the following details if this study involves the collection of plant, animal, or other materials from a natural setting:<br>• Field permit number<br>• Name of the institution or relevant body that granted permission	
**Data Availability**<br><br>Authors are required to make the data underlying their research findings fully available, without restriction.<br><br>If the manuscript reports pilot or preliminary results, the data underlying those results must be made available at the time of publication. A Data Availability	N/A - the protocol does not report results

Statement describing where the data can be found is required at submission. Your answers to this question constitute the Data Availability Statement and will be published in the article, if accepted. When legal or ethical requirements prohibit public data sharing, authors must indicate how researchers may obtain access to the data. See the PLOS Data Policy and FAQ for detailed information.<br><br>**Important:** Stating 'data available on request from the author' is not sufficient. If your data are only available upon request, select 'No' for the first question and explain your situation in the text box.<br><br>For protocols without pilot or preliminary data, authors are strongly encouraged to state how they plan to share research data from their study when it is completed or published.<br><br>Do the authors confirm that EITHER the protocol does not report results OR all data underlying the preliminary results will be made fully available without restriction?	
**Describe where the data may be found in full sentences. If you are copying our sample text, replace any instances of XXX with the appropriate details.**<br><br>• If **no pilot data are reported** in the manuscript, please enter the following: *No datasets were generated or analysed during the current study. All relevant data from this study will be made available upon study completion.*<br>• If the manuscript reports pilot data **held in a public repository**, include URLs, accession numbers or DOIs. If this information will only be available after acceptance, indicate this by ticking the box below. For example: *All XXX files are available from the XXX database*	Not applicable

(accession number(s) XXX, XXX.).<br>• If the manuscript reports pilot data contained **within the manuscript and/or Supporting Information files**, enter the following:<br>*All relevant data are within the manuscript and its Supporting Information files.*<br>• If neither of these applies but you are able to provide **details of access elsewhere**, with or without limitations, please do so. For example:<br><br>*Data cannot be shared publicly because of [XXX]. Data are available from the XXX Institutional Data Access / Ethics Committee (contact via XXX) for researchers who meet the criteria for access to confidential data.*<br><br>*The data underlying the results presented in the study are available from (include the name of the third party and contact information or URL).*<br>• This text is appropriate if the data are owned by a third party and authors do not have permission to share the data.<br><br>For study protocols with data management or sharing plans, authors are encouraged to briefly describe in their Data Availability Statement how data they generate will be made accessible when the study is completed. For example:<br>• Deidentified research data will be made publicly available when the study is completed and published.<br><br>* typeset	
Additional data availability information:	Tick here if your circumstances are not covered by the questions above and you need the journal’s help to make your data available.

Powered by Editorial Manager® and ProduXion Manager® from Aries Systems Corporation

Manuscript

Click here to access/download;Manuscript;covid 19 kap final 2021 manuscript.docx

Title: Knowledge, Attitude and Practices of COVID19 and associated vaccine hesitance among Households in the Gambia, 2021.Study protocol.
Sub title: Knowledge, Attitude and Practices of COVID19 among households of COVID19 in the Gambia.

Authors

Sainey Sanneh[1ŧ*], Sharmila Lareef-Jah[2ŧ*], Buba Darboe[3], Lamin Dibba[4], Lamin F. Manjang[1], Yorro Bah[1], Jalimory Suso[1], Phebian Sangia[1], Modou Njai[3] ,Sana M Sambou, Bakary Sanneh[5*]

Institutions
[1]Directorate of Health Research, Ministry of Health,The Gambia
[2]World Health Organization, Country Office, Kotu,The Gambia
[3]Directorate of Health Promotion and Education, Ministry of Health Bertil Herding Highway,Kotu,The Gambia
[4]Gambia Bureau of Statistics, Ministry of Finance & Economic Affairs,The Gambia
[5]National Public Health Laboratories, Ministry of Health,Bertil Herding Highway,Kotu,The Gambia

ŧ: Authors contributed equally

***: Corresponding authors**
Bakary Sanneh (BS) email:sheikbakary@yahoo.com
Sainey Sanneh (SS) email:sainey_sanneh@gmail.com
Sharmila Lareef-Jah (SL) email:lareefjahs@who.int

Abstract

The first imported confirmed case of COVID-19 was reported in The Gambia on 16th of March 2020 leading to the implementation of relevant public health interventions to prevent further importation and spread of the virus. However, by 8th November, 2021 the country had registered cumulatively 9.980 COVID-19 confirmed infection and 341 deaths. The country has develop and implemented Risk Communication and Community Engagement (RCCE) Action Plan since the declaration by WHO that COVID-19 outbreak as global public health threat and subsequent proclamation of the outbreak as a pandemic. Despite the sensitization, some Gambians are in denial and or misinformed of the existence of infection in the country. It is also evident that social distancing and other restrictions have not been adequately implemented by the citizenry. Only less 14% of the Gambian population have been vaccinated despite gross vaccine hesitance and disbelief. There is urgent need to investigate the knowledge, attitude and practices among the Gambians about preventive practices to control COVID-19. The proposed study will enrol 1200 households from seven Local Government Areas (LGAs). The findings of this study will inform the messaging and health promotion activities to better inform the population to comply and practices preventive approach especially the use of mask, getting vaccinated in order to reduce the negative impact of COVID-19 outbreak in The Gambia and thus as a prospective to returning to new normal life.

Keyword: Knowledge, Attitude, Practices, Vaccination hesitance, Outbreak, COVID19, Households, The Gambia

Background

Coronavirus disease 2019 (COVID-19) is a respiratory tract infection caused by a newly emergent coronavirus, that was first recognized in Wuhan, China, in December 2019[1]. Subsequently WHO published global outbreak which was later upgraded to an pandemic [2], [3].Genetic sequencing of the virus suggests that it is a beta-coronavirus closely linked to the SARS virus[4] A met analysis study reported that people with mild, moderate to severe COVID-19 infection might manifest symptoms and signs such as : cough, sore throat, high temperature, diarrhoea, headache, muscle or joint pain, fatigue, and loss or disturbance of sense of smell and taste [5].These researchers revealed that patients with symptoms such as cough and fever had 21% increased risk to turn COVID 19 positive. Furthermore manifestation of loss of sense of smell or taste were substantially increased the likelihood of COVID 19 infection by 8% as set of the patients reviewed[5]. It was alluded that some of the study participants were positive for COVID 19 different studies had no signs and symptoms of COVID 19 and thus blurring the fight against such a pandemic[5].In the post vaccination era of COVID 19 pandemic the pooled prevalence of 15% mortality with found to the associated in hospital admissions[6].These researchers established that patients with acute respiratory distress syndrome were eight times more likely to die as compare to those who did not have [6]. This resulted to developed of anxiety and worries on among two third of the world population as demonstrated in a study [7].Countries had implemented difference public health strategy to prevent, contain the spread of the outbreak which involve national lockdown, border closure, social distancing ,mask wearing and quarantine. The implementation of these intervention had increase change the world social norms to a new order and hence citizen had embrace and complying this situation in a difference array for instance compliance rate was at 93% for Tonga whilst 43% in Egypt among the respondents[7]

The rapid evolution of mutant variant of COVID 19 result in an increase for cause more spread and severe diseases as worsen the global response plan to curb the pandemic. Hence, the only hope to revive and re-establishes normalcy in global lifestyle was to the discovery, approval and vaccine of the global. COVID-19 vaccines were approved in late 2020 and early 2021 for public use in countries across the world and the success to this public intervention will depend not only the vaccine safety, effectiveness but coverage rate to provide hard immunity [8] .A COVID 19 vaccine hesitancy meta study revealed a varied vaccine acceptance rate in the region and country specific[8]. High acceptance were reported in Ecuador (97.0%), Malaysia (94.3%), Indonesia (93.3%) and China (91.3%);moderate acceptance Jordan (28.4%), Italy (53.7), Russia (54.9%), Poland (56.3%), US (56.9%), and France (58.9%) and lowest acceptance Kuwait (23.6%), Jordan (28.4%), from 27.7% in the Democratic Republic of the Congo Low rates of COVID-19 vaccine acceptance were reported in the Middle East, Russia, Africa and several European countries[9]. Such studies are particularly needed in the Middle East and North Africa, Sub-Saharan Africa, Eastern Europe, Central Asia, Middle and South America. Addressing the scope of COVID-19 vaccine hesitancy in various countries is recommended as an initial step for building trust in COVID-19 vaccination efforts[9].

In response to the rapid spread of the virus, the World Health Organization (WHO) declared COVID-19 a Public Health Emergency of International Concern (PHEIC) 30 January 2020 (30), and a pandemic 11 March 2020[2] [3]. The Gambia embarked on developing and implement the National preparedness and Response Plan (Response plan) [10].The Gambia registered the first case of COVID-19, 16 of March 2020 and thus the Public Health Emergency Operational Centre was operationalization to using the Incidence Management System to guideline and coordinate the national response to the outbreak [11] [12]. The country adapted and implemented of incident management plan that involves several measures to interrupt importation and transmission of the virus nationwide [13]. These include the closing of schools, suspension of public gathering, closure of all non-essential public places, spatial distancing, respiratory etiquette, restriction on number of passengers allowed on public transport and Airport closure by 17 March 2020 to the , mandatory quarantine of travelers, isolation and care for infected and suspected cases [11] [12] [13]. Massive community engagements activities were conducted to raise awareness about COVID 19 means of prevention and control practices and the provision of masks, hand sanitizers and hand wash facilities.

A toll-free helpline (1025) which was instituted during the Ebola preparation and response in 2014 was reactivated and establish and furnished call centre which operate 24 hours and 7 days by Ministry of Health[11]. These has facilitated the citizenry make inquiries on COVID-19, seek support and advice if they notice any signs and symptoms or report possible suspects or complaints regarding people defying control measures[11]. However, July 2020 most the COVID restrictions were loosened and the Airport was opened but the testing of travellers in both departing and entry the country through Airport are tested [11] [14]. The traveller accounted for 90% of the daily testing of COVID samples at the National Public Health Laboratories. The rapid test COVID 19 has been introduced and scale up nation-wide access to testing facilities. As of the 399th national situation COVID 19 report COVID-19-related death registered, bringing the total to 341 (Crude Case-Fatality Ratio, 3.4%); (Cumulative confirmed cases 9,980).The country witness intermit outbreak of COVID 19 in the following period July to September 2020 then a mild second wave in January to April 2021 and severe wave which was mainly associated with the delta variant from July to September 2021[11].COVID 19 virus lineages A and B, have been detected and associated as the cause of second wave of the national outbreak. Of which Lineage B constitutes almost 98% of the total genomes sequenced, with the sub-lineage B.1 being the most prevalent[15] whilst the third COVID outbreak wave is confirmed to be mainly associated with the transmission of the delta variant[16]. Nationally a study had phylogenetically confirmed two reinfections among healthy individuals, with a time lag of 5 months and 6 months, respectively [17].This necessitated the introduction of vaccination to increase the attainment of herd immunity and thus the introduction of the public health intervention on 15th March 2021[11].As 8th of November the national COVID 19 coverage of targeted population with completed vaccination dose was 14.2% and whilst coverage of at least one dose was 15.1%.COVID 19 vaccination coverage of the least of all ever administered vaccines in the country which could be associated with misinformation and lack of trust in the existence of the outbreak and other associated factors[11]. There is limited information to explain why people are hesitant to take the COVID 19 vaccinations despite massive community engagement activities done. Furthermore, these rumours a.re spreading through WhatsApp groups, Facebook and other electronic channels. There is an urgent need to understand the general public's awareness and perceptions on COVID-19. This is particularly important as the adherence to the control measures by the public has been viewed to be suboptimal.

The information from this survey will not only provide data for further assessments but will help to develop targeted strategies to rapidly improve current behavioural and risk communication interventions. Thus this study seek to understand the knowledge, attitude, practices associated with COVID infection and vaccination.

Hypothesis:

The Hypothesis is that the knowledge, attitude and practice of communities / household members may be average to prevent and control COVID 19 infection but have misbelief about the effectiveness and safety vaccination.

The Research Questions are:

1. What is the level of knowledge about COVID-19 and associated vaccination among household members?
2. What is the attitude of household members towards COVID-19 and associated vaccinations?
3. What are the practices of household members towards COVID-19 and associated vaccinations?

METHODOLOGY AND MATERIALS

STUDY DESIGN

A cross-sectional study design will be employed to explore the knowledge, attitude and practices of households in the context of COVID-19 prevention and control practices which includes the willingness to accept vaccination.

STUDY SITES

Enumerated areas from the seven LGAs in the country will be randomly selected. These LGAs will be further stratified as rural, urban, peri-urban areas to determine the disparity of understanding and practices of prevention and control measures of COVID-19 in the country amidst access to social amenities.

STUDY POPULATION

ELIGIBILITY CRITERIA

Inclusion criteria:

- Individuals identified for recruitment into the selected Enumeration Areas (EAs) and households in particular.

Exclusion criteria:

- Participants less than 18 years of age.

SAMPLING METHODOLOGY

Sample Design and Selection

Sample Size

Considering that the true variability of the characteristic of interest in the population is unknown in advance, sample size in this survey was computed taking into account the total number of households in the country, the sample design and method of estimation, and the response rate. Due to lack of a key

indicator (P) to be measured by the survey, a value to 50% is assumed to give the maximum level of variability. Given that it's satisfactory if the true population proportion is within ±5, an anticipated response rate of 90% was used to effectively achieve the desired precision for the estimates. Thus, the sample size for the survey is computed as follows:

$$n = \frac{deft^2 \times (1/P - 1)}{\alpha^2}$$

For the purpose of the Survey, the following assumptions inform the sample size calculation:

- P = 50% or 0.5
- Squared design effect (deft)= 1.5
- Level of Confidence = 95% i.e. α=0.05
- Margin of Error = ± 5%
- Response rate = 90% or 0.90

Using the formula and the parameters above, a minimum sample size of 900 was computed at the national level. This sample size will give a coefficient of variation (CV) which is also known as relative standard error (RSE) of 5 per cent at the national level. Adjusting the sample using a response rate of 90 per cent will give 1,000 household interviews. Given that the country is administratively divided into eight LGAs, a multi-stage stage cluster sampling will be used to select samples in three stages from the frame. In the first stage, after sorting the frame by LGA and Region (i.e. Urban and Rural), 100 EAs will be independently selected using probability proportional to household size. The figure 1 below shows a summary of the sample design.

Sample Frame

The list containing Enumeration Areas (EAs) of all the eight geographic and administrative regions in The Gambia and their respective households and population obtained from 2013 Gambia Population and Housing Census Frame will be used as the sampling frame. Consequently, with the data and cartographic information, all the EAs will be selected at the first stage based on the design proposed for this study.

Sample Design

For this study, a multi-stage stratified cluster sample design will be used to select the eligible respondents in three stages. In the first stage, after sorting the frame, EAs will be selected using Probability Proportional to the Size (PPS) of EA. As defined in the 2013 Population and Housing Census, EA size is the number of residential households residing in the EA during the population census.

Selection Scheme

In the second stage, EAs will be sorted and stand as strata in the new frame. In each of the respective selected EAs, 10 households will be selected using equal probability systematic sampling procedures. Before the data collection, a household listing operation for each of the selected EAs will be carried out by field supervisors and enumerators. All the households will be listed and ten (10) households will be

Table 1: Allocation of Enumeration Areas (EAs) to different strata						
**LGA**	**Total EAs**	**Total Urban**	**Total Rural**	**Total Selected EAs**	**Selected Urban EAs**	**Selected Rural EAs**
Banjul	74	74	-	2	2	-
Kanifing	773	773	-	22	22	-
Brikama	1,466	1,338	128	45	23	22
Kerewan	493	106	387	4	2	2
Mansakonko	204	32	172	8	5	3
Kuntaur	237	16	221	5	2	3
Janjanbureh	297	43	254	6	3	3
Basse	554	158	396	8	2	6
**Total**	**4,098**	**2,540**	**1,558**	**100**	**61**	**39**

selected using simple random sampling (third stage). The identified households to be interviewed will be visited by the field staff. Strictly, it is important to note that survey interviewer must interview only the selected households. To prevent bias, no replacements and no changes of the selected households will be permitted in the implementing stages. One individual aged 18 years and above will be selected randomly for the interview using knish grid.

Table 1 below gives the allocation of selected EAs in the various domains. The population of those 18 years and above from the 2018 Labour Force Survey was used in allocating the sample to the urban and rural strata in each LGA in order to ensure that the sample is representative of the target group.

Distribution of selected households for various LGAs is shown in Table 2.

Table 2: Allocation of Enumeration Areas (EAs) to different strata		
**LGA**	**Total Households**	**Selected Households**
Banjul	7272	20
Kanifing	69890	220
Brikama	103664	450
Kerewan	11965	40
Mansakonko	27862	80
Kuntaur	10957	50
Janjanbureh	14451	60
Basse	34641	80

**Total**	**280,702**	**1,000**

Listing procedures

All 100 selected EAs will be listed in order to update the number of households in each frame. The currently ongoing Integrated Household Survey 2020/2021 listing template will be used to obtain the total eligible households per cluster. This is a very crucial step as the computation of the second-stage probability of selection will depend entirely on the accuracy and reliability of these numbers. It is imperative to explore digital data collection this time around to enable the coordinating team ensure quality as well as select unbiased second-stage samples at the central level. Also, the coordinates of the selected structures can easily be drawn on the map for easy identification thereby saving the enumerators from identifying the wrong structures selected to participate in the survey.

Weighting procedures

By design, complex sampling involving selection at different stages will be used in the study. Inclusion probabilities will be calculated from each stage of selection and as a result, sampling weights will be computed and used for the analysis to ensure representativeness of the sample. The selection probabilities at each stage will be documented and the inverse of these probabilities is the basic weight also known as the design weight.

The household design weight is the inverse of the overall selection probability of the household which is computed as the product of the first stage (PSU/cluster) selection probability and the second stage (household) selection probability.

The number of eligible individuals per household multiplied by the corresponding household design weight in the cluster gives the individual weights.

Survey Instrument and Variables

A questionnaire was adapted to The Gambia context from the standardized IFRC, UNCIF and WHO Risk Communication and Community Engagement (RCCE) Action Plan [18] will be used for the survey(provided as supporting documents). Although it has been adapted to the local context, the questionnaire will be pretested and findings will be used to further refine the questions. The survey items are organised in four sections namely demographics, knowledge, attitudes and practices and consists of primarily close-ended questions. Consent will be obtained before administration of the questionnaire.

Data Management, Analysis and Presentation

Data collection will be done by trained and experienced enumerators. A virtual meeting with enumerators will be held daily to review the process and identify challenges and successes. An exploratory data analysis (EDA) will be conducted after the collection of the data in order to make a quick check into the consistency and validity of the data. Subsequently, necessary data cleaning will be done to prepare the data for analysis. Analysis of the data collected will be conducted using the International Business Machines Statistical Package for Social Sciences (IBM SPSS). Frequencies will be run to explore missing responses and out-of-range values for each of the demographic variables as

well as those used for the main analysis. The distribution of the data will be subjected to normality test using the Shapiro-Wilk test of normality. As the data collection is premised on assessing the knowledge, attitude and practices towards Covid-19 and willingness to accept vaccination, analysis will focus on key demographic variables such as gender, location and age. Contingency tables will be constructed to look at the relationship between key demographic variables and selected KAP variables of interest.

The analysis of the data will focus on the three components of the survey namely knowledge, attitude and practice and association with willingness to accept COVID vaccinations. Knowledge assesses the amount of knowledge respondents have about COVID19 and the source(s) of information and associated vaccination belief. Attitude questions will address people's attitude towards the pandemic such as their belief that it is a reality while practice questions will focus on the activities people are engaged in in preventing themselves, their families and their neighbours from the disease through vaccination and other practices. Key results will be presented in the form of tables and graphs and the necessary statistical interpretations will be done in the form of an analytical report for policy makers. The study finding will be presented stakeholders, partners in COVID 19 response.

Potential Risks

There is anticipated risk of infection to the public or data collectors given that we are in an active pandemic. Therefore to minimise such risk all data collectors and drivers will be trained on basic IPC procedures, must be vaccinated. In addition, interviews will be conducted maintaining the WHO-recommended social distancing guidelines, and compulsory wearing of face mask.

Ethical Consideration

The study has been approved The Gambia Government/MRCG Laboratories Joint Ethics Committee (Project ID/ethics ref: 22699) and from the Ministry of Health for the study protocol and procedures of informed consent. Informed consent of all participants will be sought by explaining purpose of the study to them in the language they best understand. Information from participants will be treated confidentially and will not be shared with anyone except study team members.

Expected outcome and implication of findings

This survey will reveal an accurate picture of what the general population knows about the pandemic and their attitudes and practices towards prevention and control measures for this disease and their willingness to accept vaccination. It will assess the huge investment the government and partners and donors had facilitate the implementation of the Communication and Community Engagement (RCCE) Action Plan. This will help to guide the review and updating RCCE plan to impact better behavioural change toward eventually positive acceptance and practices prevention and control of COVID19 and subsequent increase for vaccination coverage which will assure the attainment of herd immunity.

Acknowledgement

The authors appreciated the review and inputs of the Gambia Government and MRC Joint Ethics Committee.

Conflict of interest

The authors have declared no conflict of interest

Funding sources

The study protocol will be funded by WHO, The Gambia Country.

Disclaimer: The involvement of WHO country office staff does not influence the study design and represent the office in the study.

REFERENCES

1. WHO, Report of the WHO-China Joint Mission on Coronavirus Disease 2019 (COVID-19). 2020.
2. World Health Organization. The 2019-nCoV outbreak is an emergency of international concern. 2020.
3. World Health Organization. WHO Director-General’s opening remarks at the media briefing on COVID-19-11 March 2020.
4. Lu R, Zhao X, Li J, Niu P, Yang B, Wu H, et al. Genomic characterisation and epidemiology of 2019 novel coronavirus: implications for virus origins and receptor binding. The lancet. 2020 Feb 22;395(10224):565-74..
5. Struyf T, Deeks JJ, Dinnes J, Takwoingi Y, Davenport C, Leeflang MM, et al. Signs and symptoms to determine if a patient presenting in primary care or hospital outpatient settings has COVID- 19. Cochrane Database of Systematic Reviews. 2021(2).
6. Abate SM, Checkol YA, Mantefardo B. Global prevalence and determinants of mortality among patients with COVID-19: A systematic review and meta-analysis. Annals of Medicine and Surgery. 2021 Mar 4:102204.
7. Mannan DK, Farhana KM. Knowledge, attitude and acceptance of a COVID-19 vaccine: A global cross-sectional study. International Research Journal of Business and Social Science. 2020 Dec 7; 6(4).
8. Biswas N, Mustapha T, Khubchandani J, Price JH. The Nature and Extent of COVID-19 Vaccination Hesitancy in Healthcare Workers. Journal of Community Health. 2021 Apr 20:1-8
9. Sallam M. COVID-19 vaccine hesitancy worldwide: a concise systematic review of vaccine acceptance rates. Vaccines. 2021 Feb; 9(2):160.
10. The Republic the Gambia, National Novel Coronavirus (COVID-19) Preparedness and Response Plan. 2020 April, VOL-1 VERSION-02.

11. Gambia-COVID-19-Situational –Report_2021_7th_8th_November_No-399
12. The Republic of the Gambia, Ministry of Health Public Health Emergency Operation Centre guideline,2019
13. Nabaneh S. The Use of Emergency Powers in Response to COVID-19 in the Gambia. Verfassungsblog: On Matters Constitutional. 2021 May 6.
14. Jallow R. Challenges of The Gambia's COVID-19 response and policy recommendations. Cities & Health. 2020 Nov 4:1-5.
15. Kanteh A, Manneh J, Jabang S, Kujabi MA, Sanyang B, Oboh MA,et al. Origin of imported SARS-CoV-2 strains in The Gambia identified from whole genome sequences. PloS one. 2021 Aug 31;16(8):e0241942.
16. Statement by UN System in The Gambia on the detection of COVID 19 Delta variant in the country 01 June 2021.https://www.unicef.org/gambia/press-releases/statement-un-system-gambia-recent-detection-covid-19-delta-variant-country.
17. Sanyang B, Kanteh A, Usuf E, Nadjm B, Jarju S, Bah A,et al. COVID-19 reinfections in The Gambia by phylogenetically distinct SARS-CoV-2 variants—first two confirmed events in West Africa. The Lancet Global Health. 2021 Jun 3.
18. World Health Organization. COVID-19 global risk communication and community engagement strategy, December 2020-May 2021: interim guidance, 23 December 2020. World Health Organization; 2020.

Support documents

Figure 1. Sampling framework

Manuscript
Click here to access/download;Manuscript;covid 19 kap final 2021 manuscript_Revised version 1.docx

Title: Knowledge, Attitude and Practices of COVID19 and associated vaccine hesitance among Households in the Gambia, 2021.Study protocol.
Sub title: Knowledge, Attitude and Practices of COVID19 among households of COVID19 in the Gambia.

Authors

Sainey Sanneh[1ŧ*], Sharmila Lareef-Jah[2ŧ*], Buba Darboe[3], Lamin Dibba[4], Lamin F. Manjang[1], Yorro Bah[1], Jalimory Suso[1], Phebian Sangia[1], Modou Njai[3] ,Sana M Sambou, Bakary Sanneh[5*]

Institutions

[1]Directorate of Health Research, Ministry of Health,The Gambia
[2]World Health Organization, Country Office, Kotu,The Gambia
[3]Directorate of Health Promotion and Education, Ministry of Health Bertil Herding Highway,Kotu,The Gambia
[4]Gambia Bureau of Statistics, Ministry of Finance & Economic Affairs,The Gambia
[5]National Public Health Laboratories, Ministry of Health,Bertil Herding Highway,Kotu,The Gambia

ŧ: Authors contributed equally

***: Corresponding authors**

Bakary Sanneh (BS) email:sheikbakary@yahoo.com
Sainey Sanneh (SS) email:sainey_sanneh@gmail.com
Sharmila Lareef-Jah (SL) email:lareefjahs@who.int

Abstract

The first imported confirmed case of COVID-19 was reported in The Gambia on 16th of March 2020 leading to the implementation of relevant public health interventions to prevent further importation and spread of the virus. However, by 8th November, 2021 the country had registered cumulatively 9.980 COVID-19 confirmed infection and 341 deaths. The country has develop and implemented Risk Communication and Community Engagement (RCCE) Action Plan since the declaration by WHO that COVID-19 outbreak as global public health threat and subsequent proclamation of the outbreak as a pandemic. Despite the sensitization, some Gambians are in denial and or misinformed of the existence of infection in the country. It is also evident that social distancing and other restrictions have not been adequately implemented by the citizenry. Only less 14% of the Gambian population have been vaccinated despite gross vaccine hesitance and disbelief. There is urgent need to investigate the knowledge, attitude and practices among the Gambians about preventive practices to control COVID-19. The proposed study will enrol 1200 households from seven Local Government Areas (LGAs). The findings of this study will inform the messaging and health promotion activities to better inform the population to comply and practices preventive approach especially the use of mask, getting vaccinated in order to reduce the negative impact of COVID-19 outbreak in The Gambia and thus as a prospective to returning to new normal life.

Keyword: Knowledge, Attitude, Practices, Vaccination hesitance, Outbreak, COVID19, Households, The Gambia

Background

Coronavirus disease 2019 (COVID-19) is a respiratory tract infection caused by a newly emergent coronavirus, that was first recognized in Wuhan, China, in December 2019[1]. Subsequently WHO published global outbreak which was later upgraded to an pandemic [2], [3].Genetic sequencing of the virus suggests that it is a beta-coronavirus closely linked to the SARS virus[4] A met analysis study reported that people with mild, moderate to severe COVID-19 infection might manifest symptoms and signs such as : cough, sore throat, high temperature, diarrhoea, headache, muscle or joint pain, fatigue, and loss or disturbance of sense of smell and taste [5].These researchers revealed that patients with symptoms such as cough and fever had 21% increased risk to turn COVID 19 positive. Furthermore manifestation of loss of sense of smell or taste were substantially increased the likelihood of COVID 19 infection by 8% as set of the patients reviewed[5]. It was alluded that some of the study participants were positive for COVID 19 different studies had no signs and symptoms of COVID 19 and thus blurring the fight against such a pandemic[5].In the post vaccination era of COVID 19 pandemic the pooled prevalence of 15% mortality with found to the associated in hospital admissions[6].These researchers established that patients with acute respiratory distress syndrome were eight times more likely to die as compare to those who did not have [6]. This resulted to developed of anxiety and worries on among two third of the world population as demonstrated in a study [7].Countries had implemented difference public health strategy to prevent, contain the spread of the outbreak which involve national lockdown, border closure, social distancing ,mask wearing and quarantine. The implementation of these intervention had increase change the world social norms to a new order and hence citizen had embrace and complying this situation in a difference array for instance compliance rate was at 93% for Tonga whilst 43% in Egypt among the respondents[7]

The rapid evolution of mutant variant of COVID 19 result in an increase for cause more spread and severe diseases as worsen the global response plan to curb the pandemic. Hence, the only hope to revive and re-establishes normalcy in global lifestyle was to the discovery, approval and vaccine of the global. COVID-19 vaccines were approved in late 2020 and early 2021 for public use in countries across the world and the success to this public intervention will depend not only the vaccine safety, effectiveness but coverage rate to provide hard immunity [8] .A COVID 19 vaccine hesitancy meta study revealed a varied vaccine acceptance rate in the region and country specific[8]. High acceptance were reported in Ecuador (97.0%), Malaysia (94.3%), Indonesia (93.3%) and China (91.3%);moderate acceptance Jordan (28.4%), Italy (53.7), Russia (54.9%), Poland (56.3%), US (56.9%), and France (58.9%) and lowest acceptance Kuwait (23.6%), Jordan (28.4%), from 27.7% in the Democratic Republic of the Congo Low rates of COVID-19 vaccine acceptance were reported in the Middle East, Russia, Africa and several European countries[9]. Such studies are particularly needed in the Middle East and North Africa, Sub-Saharan Africa, Eastern Europe, Central Asia, Middle and South America. Addressing the scope of COVID-19 vaccine hesitancy in various countries is recommended as an initial step for building trust in COVID-19 vaccination efforts[9].

In response to the rapid spread of the virus, the World Health Organization (WHO) declared COVID-19 a Public Health Emergency of International Concern (PHEIC) 30 January 2020 (30), and a pandemic 11 March 2020[2] [3]. The Gambia embarked on developing and implement the National preparedness and Response Plan (Response plan) [10].The Gambia registered the first case of COVID-19, 16 of March 2020 and thus the Public Health Emergency Operational Centre was operationalization to using the Incidence Management System to guideline and coordinate the national response to the outbreak [11] [12]. The country adapted and implemented of incident management plan that involves several measures to interrupt importation and transmission of the virus nationwide [13]. These include the closing of schools, suspension of public gathering, closure of all non-essential public places, spatial distancing, respiratory etiquette, restriction on number of passengers allowed on public transport and Airport closure by 17 March 2020 to the , mandatory quarantine of travelers, isolation and care for infected and suspected cases [11] [12] [13]. Massive community engagements activities were conducted to raise awareness about COVID 19 means of prevention and control practices and the provision of masks, hand sanitizers and hand wash facilities.

A toll-free helpline (1025) which was instituted during the Ebola preparation and response in 2014 was reactivated and establish and furnished call centre which operate 24 hours and 7 days by Ministry of Health[11]. These has facilitated the citizenry make inquiries on COVID-19, seek support and advice if they notice any signs and symptoms or report possible suspects or complaints regarding people defying control measures[11]. However, July 2020 most the COVID restrictions were loosened and the Airport was opened but the testing of travellers in both departing and entry the country through Airport are tested [11] [14]. The traveller accounted for 90% of the daily testing of COVID samples at the National Public Health Laboratories. The rapid test COVID 19 has been introduced and scale up nation-wide access to testing facilities. As of the 399th national situation COVID 19 report COVID-19-related death registered, bringing the total to 341 (Crude Case-Fatality Ratio, 3.4%); (Cumulative confirmed cases 9,980).The country witness intermit outbreak of COVID 19 in the following period July to September 2020 then a mild second wave in January to April 2021 and severe wave which was mainly associated with the delta variant from July to September 2021[11].COVID 19 virus lineages A and B, have been detected and associated as the cause of second wave of the national outbreak. Of which Lineage B constitutes almost 98% of the total genomes sequenced, with the sub-lineage B.1 being the most prevalent[15] whilst the third COVID outbreak wave is confirmed to be mainly associated with the transmission of the delta variant[16]. Nationally a study had phylogenetically confirmed two reinfections among healthy individuals, with a time lag of 5 months and 6 months, respectively [17].This necessitated the introduction of vaccination to increase the attainment of herd immunity and thus the introduction of the public health intervention on 15th March 2021[11].As 8th of November the national COVID 19 coverage of targeted population with completed vaccination dose was 14.2% and whilst coverage of at least one dose was 15.1%.COVID 19 vaccination coverage of the least of all ever administered vaccines in the country which could be associated with misinformation and lack of trust in the existence of the outbreak and other associated factors[11]. There is limited information to explain why people are hesitant to take the COVID 19 vaccinations despite massive community engagement activities done. Furthermore, these rumours a.re spreading through WhatsApp groups, Facebook and other electronic channels. There is an urgent need to understand the general public's awareness and perceptions on COVID-19. This is particularly important as the adherence to the control measures by the public has been viewed to be suboptimal.

The information from this survey will not only provide data for further assessments but will help to develop targeted strategies to rapidly improve current behavioural and risk communication interventions. Thus this study seek to understand the knowledge, attitude, practices associated with COVID infection and vaccination.

Hypothesis:

The Hypothesis is that the knowledge, attitude and practice of communities / household members may be average to prevent and control COVID 19 infection but have misbelief about the effectiveness and safety vaccination.

The Research Questions are:

1. What is the level of knowledge about COVID-19 and associated vaccination among household members?
2. What is the attitude of household members towards COVID-19 and associated vaccinations?
3. What are the practices of household members towards COVID-19 and associated vaccinations?

METHODOLOGY AND MATERIALS

STUDY DESIGN

A cross-sectional study design will be employed to explore the knowledge, attitude and practices of households in the context of COVID-19 prevention and control practices which includes the willingness to accept vaccination.

STUDY SITES

Enumerated areas from the seven LGAs in the country will be randomly selected. These LGAs will be further stratified as rural, urban, peri-urban areas to determine the disparity of understanding and practices of prevention and control measures of COVID-19 in the country amidst access to social amenities.

STUDY POPULATION

ELIGIBILITY CRITERIA

Inclusion criteria:

- Individuals identified for recruitment into the selected Enumeration Areas (EAs) and households in particular.

Exclusion criteria:

- Participants less than 18 years of age.

SAMPLING METHODOLOGY

Sample Design and Selection

Sample Size

Considering that the true variability of the characteristic of interest in the population is unknown in advance, sample size in this survey was computed taking into account the total number of households in the country, the sample design and method of estimation, and the response rate. Due to lack of a key

indicator (P) to be measured by the survey, a value to 50% is assumed to give the maximum level of variability. Given that it's satisfactory if the true population proportion is within ±5, an anticipated response rate of 90% was used to effectively achieve the desired precision for the estimates. Thus, the sample size for the survey is computed as follows:

$$n = \frac{deft^2 \times (1/P - 1)}{\alpha^2}$$

For the purpose of the Survey, the following assumptions inform the sample size calculation:

- P = 50% or 0.5
- Squared design effect (deft)= 1.5
- Level of Confidence = 95% i.e. α=0.05
- Margin of Error = ± 5%
- Response rate = 90% or 0.90

Using the formula and the parameters above, a minimum sample size of 900 was computed at the national level. This sample size will give a coefficient of variation (CV) which is also known as relative standard error (RSE) of 5 per cent at the national level. Adjusting the sample using a response rate of 90 per cent will give 1,000 household interviews. Given that the country is administratively divided into eight LGAs, a multi-stage stage cluster sampling will be used to select samples in three stages from the frame. In the first stage, after sorting the frame by LGA and Region (i.e. Urban and Rural), 100 EAs will be independently selected using probability proportional to household size. The figure 1 below shows a summary of the sample design.

Sample Frame

The list containing Enumeration Areas (EAs) of all the eight geographic and administrative regions in The Gambia and their respective households and population obtained from 2013 Gambia Population and Housing Census Frame will be used as the sampling frame. Consequently, with the data and cartographic information, all the EAs will be selected at the first stage based on the design proposed for this study.

Sample Design

For this study, a multi-stage stratified cluster sample design will be used to select the eligible respondents in three stages. In the first stage, after sorting the frame, EAs will be selected using Probability Proportional to the Size (PPS) of EA. As defined in the 2013 Population and Housing Census, EA size is the number of residential households residing in the EA during the population census.

Selection Scheme

In the second stage, EAs will be sorted and stand as strata in the new frame. In each of the respective selected EAs, 10 households will be selected using equal probability systematic sampling procedures. Before the data collection, a household listing operation for each of the selected EAs will be carried out by field supervisors and enumerators. All the households will be listed and ten (10) households will be

Table 1: Allocation of Enumeration Areas (EAs) to different strata						
**LGA**	**Total EAs**	**Total Urban**	**Total Rural**	**Total Selected EAs**	**Selected Urban EAs**	**Selected Rural EAs**
Banjul	74	74	-	2	2	-
Kanifing	773	773	-	22	22	-
Brikama	1,466	1,338	128	45	23	22
Kerewan	493	106	387	4	2	2
Mansakonko	204	32	172	8	5	3
Kuntaur	237	16	221	5	2	3
Janjanbureh	297	43	254	6	3	3
Basse	554	158	396	8	2	6
**Total**	**4,098**	**2,540**	**1,558**	**100**	**61**	**39**

selected using simple random sampling (third stage). The identified households to be interviewed will be visited by the field staff. Strictly, it is important to note that survey interviewer must interview only the selected households. To prevent bias, no replacements and no changes of the selected households will be permitted in the implementing stages. One individual aged 18 years and above will be selected randomly for the interview using knish grid.

Table 1 below gives the allocation of selected EAs in the various domains. The population of those 18 years and above from the 2018 Labour Force Survey was used in allocating the sample to the urban and rural strata in each LGA in order to ensure that the sample is representative of the target group.

Distribution of selected households for various LGAs is shown in Table 2.

Table 2: Allocation of Enumeration Areas (EAs) to different strata		
**LGA**	**Total Households**	**Selected Households**
Banjul	7272	20
Kanifing	69890	220
Brikama	103664	450
Kerewan	11965	40
Mansakonko	27862	80
Kuntaur	10957	50
Janjanbureh	14451	60
Basse	34641	80

**Total**	**280,702**	**1,000**

Listing procedures

All 100 selected EAs will be listed in order to update the number of households in each frame. The currently ongoing Integrated Household Survey 2020/2021 listing template will be used to obtain the total eligible households per cluster. This is a very crucial step as the computation of the second-stage probability of selection will depend entirely on the accuracy and reliability of these numbers. It is imperative to explore digital data collection this time around to enable the coordinating team ensure quality as well as select unbiased second-stage samples at the central level. Also, the coordinates of the selected structures can easily be drawn on the map for easy identification thereby saving the enumerators from identifying the wrong structures selected to participate in the survey.

Weighting procedures

By design, complex sampling involving selection at different stages will be used in the study. Inclusion probabilities will be calculated from each stage of selection and as a result, sampling weights will be computed and used for the analysis to ensure representativeness of the sample. The selection probabilities at each stage will be documented and the inverse of these probabilities is the basic weight also known as the design weight.

The household design weight is the inverse of the overall selection probability of the household which is computed as the product of the first stage (PSU/cluster) selection probability and the second stage (household) selection probability.

The number of eligible individuals per household multiplied by the corresponding household design weight in the cluster gives the individual weights.

Survey Instrument and Variables

A questionnaire was adapted to The Gambia context from the standardized IFRC, UNCIF and WHO Risk Communication and Community Engagement (RCCE) Action Plan [18] will be used for the survey(provided as supporting documents). Although it has been adapted to the local context, the questionnaire will be pretested and findings will be used to further refine the questions. The survey items are organised in four sections namely demographics, knowledge, attitudes and practices and consists of primarily close-ended questions. Consent will be obtained before administration of the questionnaire.

Data Management, Analysis and Presentation

Data collection will be done by trained and experienced enumerators. A virtual meeting with enumerators will be held daily to review the process and identify challenges and successes. An exploratory data analysis (EDA) will be conducted after the collection of the data in order to make a quick check into the consistency and validity of the data. Subsequently, necessary data cleaning will be done to prepare the data for analysis. Analysis of the data collected will be conducted using the International Business Machines Statistical Package for Social Sciences (IBM SPSS). Frequencies will be run to explore missing responses and out-of-range values for each of the demographic variables as

well as those used for the main analysis. The distribution of the data will be subjected to normality test using the Shapiro-Wilk test of normality. As the data collection is premised on assessing the knowledge, attitude and practices towards Covid-19 and willingness to accept vaccination, analysis will focus on key demographic variables such as gender, location and age. Contingency tables will be constructed to look at the relationship between key demographic variables and selected KAP variables of interest.
The analysis of the data will focus on the three components of the survey namely knowledge, attitude and practice and association with willingness to accept COVID vaccinations. Knowledge assesses the amount of knowledge respondents have about COVID19 and the source(s) of information and associated vaccination belief. Attitude questions will address people's attitude towards the pandemic such as their belief that it is a reality while practice questions will focus on the activities people are engaged in in preventing themselves, their families and their neighbours from the disease through vaccination and other practices. Key results will be presented in the form of tables and graphs and the necessary statistical interpretations will be done in the form of an analytical report for policy makers. The study finding will be presented stakeholders, partners in COVID 19 response.

Potential Risks

There is anticipated risk of infection to the public or data collectors given that we are in an active pandemic. Therefore to minimise such risk all data collectors and drivers will be trained on basic IPC procedures, must be vaccinated. In addition, interviews will be conducted maintaining the WHO-recommended social distancing guidelines, and compulsory wearing of face mask.

Ethical Consideration

The study has been approved The Gambia Government/MRCG Laboratories Joint Ethics Committee (Project ID/ethics ref: 22699) and from the Ministry of Health for the study protocol and procedures of informed consent. Written informed consent will be obtained from all participants. The participants will be provided with adequate information about purpose of the study in a language they best understand. Information from participants will be treated confidentially and will not be shared with anyone except study team members.

Expected outcome and implication of findings

This survey will reveal an accurate picture of what the general population knows about the pandemic and their attitudes and practices towards prevention and control measures for this disease and their willingness to accept vaccination. It will assess the huge investment the government and partners and donors had facilitate the implementation of the Communication and Community Engagement (RCCE) Action Plan. This will help to guide the review and updating RCCE plan to impact better behavioural change toward eventually positive acceptance and practices prevention and control of COVID19 and subsequent increase for vaccination coverage which will assure the attainment of herd immunity.

Acknowledgement
The authors appreciated the review and inputs of the Gambia Government and MRC Joint Ethics Committee.

Conflict of interest
The authors have declared no conflict of interest

Funding sources
The study protocol will be funded by WHO, The Gambia Country.

Disclaimer: The involvement of WHO country office staff does not influence the study design and represent the office in the study.

REFERENCES

1. WHO, Report of the WHO-China Joint Mission on Coronavirus Disease 2019 (COVID-19). 2020.
2. World Health Organization. The 2019-nCoV outbreak is an emergency of international concern. 2020.
3. World Health Organization. WHO Director-General’s opening remarks at the media briefing on COVID-19-11 March 2020.
4. Lu R, Zhao X, Li J, Niu P, Yang B, Wu H, et al. Genomic characterisation and epidemiology of 2019 novel coronavirus: implications for virus origins and receptor binding. The lancet. 2020 Feb 22;395(10224):565-74..
5. Struyf T, Deeks JJ, Dinnes J, Takwoingi Y, Davenport C, Leeflang MM, et al. Signs and symptoms to determine if a patient presenting in primary care or hospital outpatient settings has COVID- 19. Cochrane Database of Systematic Reviews. 2021(2).
6. Abate SM, Checkol YA, Mantefardo B. Global prevalence and determinants of mortality among patients with COVID-19: A systematic review and meta-analysis. Annals of Medicine and Surgery. 2021 Mar 4:102204.
7. Mannan DK, Farhana KM. Knowledge, attitude and acceptance of a COVID-19 vaccine: A global cross-sectional study. International Research Journal of Business and Social Science. 2020 Dec 7; 6(4).
8. Biswas N, Mustapha T, Khubchandani J, Price JH. The Nature and Extent of COVID-19 Vaccination Hesitancy in Healthcare Workers. Journal of Community Health. 2021 Apr 20:1-8
9. Sallam M. COVID-19 vaccine hesitancy worldwide: a concise systematic review of vaccine acceptance rates. Vaccines. 2021 Feb; 9(2):160.

10. The Republic the Gambia, National Novel Coronavirus (COVID-19) Preparedness and Response Plan. 2020 April, VOL-1 VERSION-02.
11. Gambia-COVID-19-Situational –Report_2021_7th_8th_November_No-399
12. The Republic of the Gambia, Ministry of Health Public Health Emergency Operation Centre guideline,2019
13. Nabaneh S. The Use of Emergency Powers in Response to COVID-19 in the Gambia. Verfassungsblog: On Matters Constitutional. 2021 May 6.
14. Jallow R. Challenges of The Gambia's COVID-19 response and policy recommendations. Cities & Health. 2020 Nov 4:1-5.
15. Kanteh A, Manneh J, Jabang S, Kujabi MA, Sanyang B, Oboh MA,et al. Origin of imported SARS-CoV-2 strains in The Gambia identified from whole genome sequences. PloS one. 2021 Aug 31;16(8):e0241942.
16. Statement by UN System in The Gambia on the detection of COVID 19 Delta variant in the country 01 June 2021.https://www.unicef.org/gambia/press-releases/statement-un-system-gambia-recent-detection-covid-19-delta-variant-country.
17. Sanyang B, Kanteh A, Usuf E, Nadjm B, Jarju S, Bah A,et al. COVID-19 reinfections in The Gambia by phylogenetically distinct SARS-CoV-2 variants—first two confirmed events in West Africa. The Lancet Global Health. 2021 Jun 3.
18. World Health Organization. COVID-19 global risk communication and community engagement strategy, December 2020-May 2021: interim guidance, 23 December 2020. World Health Organization; 2020.

Support documents

Figure 1. Sampling framework

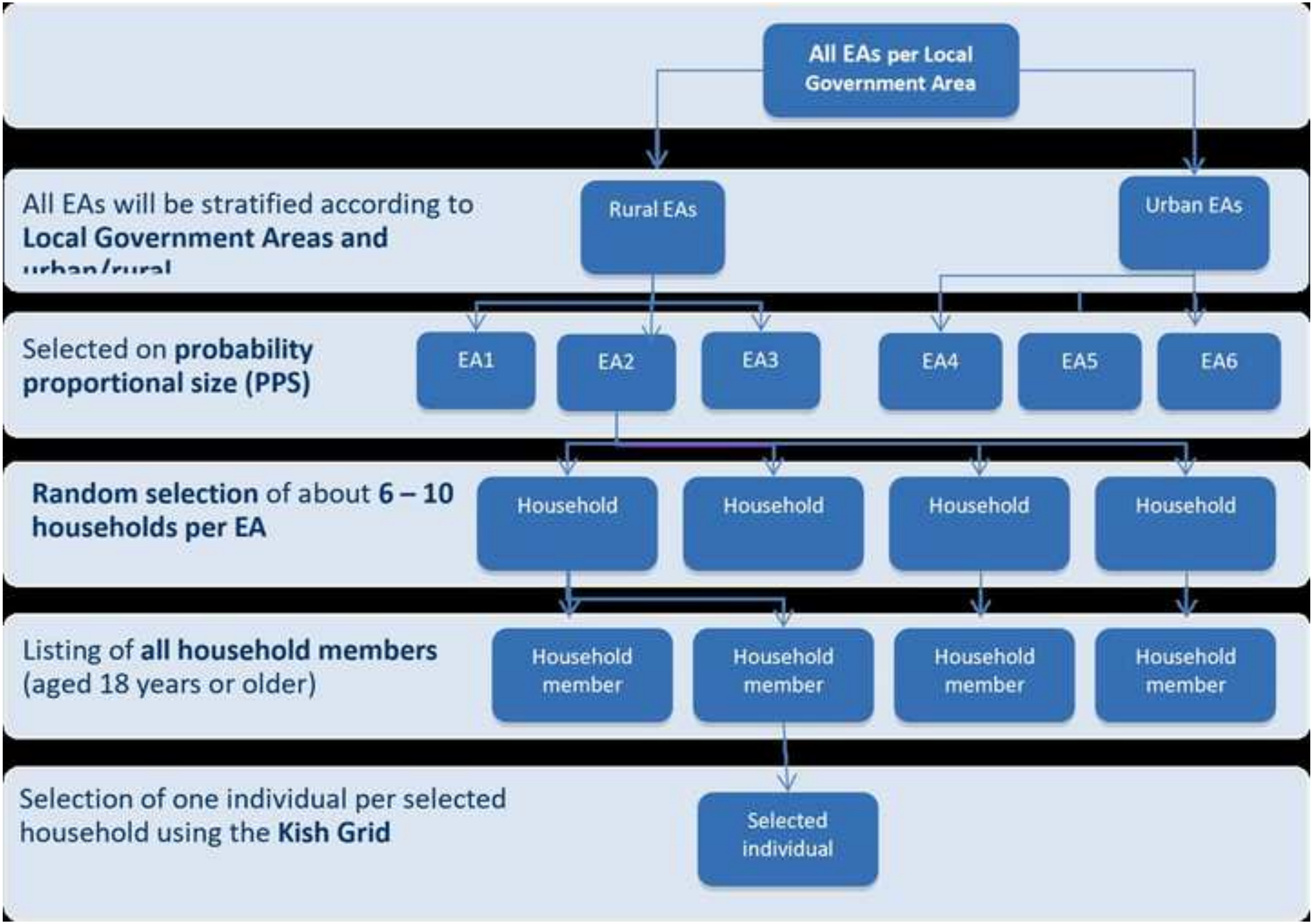
All EAs per Local Government Area
All EAs will be stratified according to Local Government Areas and
Rural EAs
Urban EAs
Selected on probability proportional size (PPS)
EA1
EA2
EA3
EA4
EA5
EA6
Random selection of about 6 – 10 households per EA
Household
Household
Household
Household
Listing of all household members (aged 18 years or older)
Household member
Household member
Household member
Household member
Selection of one individual per selected household using the Kish Grid
Selected individual

Click here to access/download
Supporting Information
Covid 19 kap Information Sheet & Consent form.docx

THE REPUBLIC OF THE GAMBIA

National Public Health Laboratories
Ministry of Health & Social Welfare
Bertil Herding Highway, Kotu Layout
(Same complex as Central Medical Stores)

National Public Health Laboratory Services
Service, quality, Health, Research, Education, Surveillance

Tel: 4466799 **info@moh.gov.gm** **Fax: 4414431**

Ref: NPHL/Adm/2021/204 **17th November 2021**

To

Editor Plos One

Dear Sir/Madam,
Re: (PONE-D-21-36320)Knowledge, Attitude and Practices of COVID19 and associated vaccine hesitance among Households in the Gambia, 2021.Study protocol.

We are indeed happy the rapid insightful review and comments. We have attempted to response to all the raised issues and updated the manuscript according and provided attachment of required documents where applicable. These are as follows:

1. We note you did not include a copy of the original, (signed or stamped) dated IRB/research ethics committee approval letter or waiver for all study sites as an 'Other' file. In keeping with our submission guidelines, please upload the approval letter or waiver as an 'Other' file. More information related to the relevant journal policies is available from: [https://journals.plos.org/plosone/s/submission-guidelines#loc-guidelines-for-specific-study-types]

Response:We have provided and uploaded the Gambia Government and MRCG Joint Ethics Committee approval

2. We note you did not include a letter from an external funder confirming the funding award you received as an 'Other' file. In keeping with our submission guidelines, please upload the Funding letter you received as an 'Other' file. If you are unable to secure a letter from the funder confirming your funding award, please note this in your cover letter. More information related to the relevant journal policies is available from: [https://journals.plos.org/plosone/s/submission-guidelines#loc-guidelines-for-specific-study-types]

Response: World Health Country office the Gambia have provided work plan funding number is as AFGMB2018552, task 6.1. These relevant information has been updated on the journal manuscript submission interface.

3. Please provide additional details regarding participant consent. In the ethics statement in the Methods and online submission information, please ensure that you have specified what type you obtained (for instance, written or verbal, and if verbal, how it was documented and witnessed). If your study included minors, state whether you obtained consent from parents or guardians. If the need for consent was waived by the ethics committee, please include this information.

Response:Written consent approach will be applied in this study and has been updated in the revised manscript section on ethic.The consent has been joint with the participant information form has been upload with the ethic approval letter in a zip folder name other.

Thanks

Bakary Sanneh
Corresponding Author,
Ag.Deputy Director-National Public Health Laboratories
National Public Health Laboratories,
Bertil Herding Highway, Kotu
Ministry of Health
Bertil Herding Highway, Kotu
e-mail: sheikbakary@yahoo.com
