# Supplementary material for: Practice of preventive measures and vaccine hesitance for COVID 19 among households in The Gambia, 2021: Study protocol
Source: PLoS One. 2022 Aug 30;17(8):e0270304. doi: 10.1371/journal.pone.0270304 (PMC9426907; doi:10.1371/journal.pone.0270304)
Supplement: Attachment — Submitted filename: Response letter_COVID KAP_2021.doc [file pone.0270304.s003.doc]

**Ref: NPHL/Adm/2021/204	17^th^ November 2021**

To

**Editor Plos One**

**Dear Sir/Madam,**

# **Re: (** PONE-D-21-36320 )Knowledge, Attitude and Practices of COVID19 and associated vaccine hesitance among Households in the Gambia, 2021.Study protocol.

We are indeed happy the rapid insightful review and comments. We have attempted to response to all the raised issues and updated the manuscript according and provided attachment of required documents where applicable. These are as follows:

1. We note you did not include a copy of the original, (signed or stamped) dated IRB/research ethics committee approval letter or waiver for all study sites as an ‘Other’ file. In keeping with our submission guidelines, please upload the approval letter or waiver as an ‘Other’ file. More information related to the relevant journal policies is available from: [[https://journals.plos.org/plosone/s/submission-guidelines#loc-guidelines-for-specific-study-types](https://journals.plos.org/plosone/s/submission-guidelines#loc-guidelines-for-specific-study-types)]

Response:We have provided and uploaded the Gambia Government and MRCG Joint Ethics Committee approval

2. We note you did not include a letter from an external funder confirming the funding award you received as an ‘Other’ file. In keeping with our submission guidelines, please upload the Funding letter you received as an ‘Other’ file. If you are unable to secure a letter from the funder confirming your funding award, please note this in your cover letter. More information related to the relevant journal policies is available from: [[https://journals.plos.org/plosone/s/submission-guidelines#loc-guidelines-for-specific-study-types](https://journals.plos.org/plosone/s/submission-guidelines#loc-guidelines-for-specific-study-types)]

Response: World Health Country office the Gambia have provided work plan funding number is as AFGMB2018552, task 6.1. These relevant information has been updated on the journal manuscript submission interface.

3. Please provide additional details regarding participant consent. In the ethics statement in the Methods and online submission information, please ensure that you have specified what type you obtained (for instance, written or verbal, and if verbal, how it was documented and witnessed). If your study included minors, state whether you obtained consent from parents or guardians. If the need for consent was waived by the ethics committee, please include this information.

Response:Written consent approach will be applied in this study and has been updated in the revised manscript section on ethic.The consent has been joint with the participant information form has been upload with the ethic approval letter in a zip folder name other.

Thanks

Bakary Sanneh

Corresponding Author,

Ag.Deputy Director-National Public Health Laboratories

National Public Health Laboratories,

Bertil Herding Highway, Kotu

Ministry of Health

Bertil Herding Highway, Kotu

e-mail: sheikbakary@yahoo.com
