# Supplementary material for: Practice of preventive measures and vaccine hesitance for COVID 19 among households in The Gambia, 2021: Study protocol
Source: PLoS One. 2022 Aug 30;17(8):e0270304. doi: 10.1371/journal.pone.0270304 (PMC9426907; doi:10.1371/journal.pone.0270304)
Supplement: Attachment — Submitted filename: Response letter_COVID KAP_30 11 V 3.doc [file pone.0270304.s005.doc]

**Ref: NPHL/Adm/2021/215	30^th^ November 2021**

To

**Editor Plos One**

**Dear Sir/Madam,**

# **Re: (** PONE-D-21-36320 )Knowledge, Attitude and Practices of COVID19 and associated vaccine hesitance among Households in the Gambia, 2021.Study protocol.

We are indeed happy the rapid insightful review and comments. We have attempted to response to all the raised issues and updated the manuscript according and provided attachment of required documents where applicable. These are as follows:

Reviewer #2: Topic selection of this research is very relevant to this current senario but, objectives of this study is not well defined. Research hypothesis is well expalined. Methodology part is well described. Regarding the analysis part, how to show the association between knowledge, attitude and practice is not explained properly.
Overall content is nice with some spelling mistakes and some gramatical errors. There is also some errors in citation part of article.

Response:The grammatical errors were corrected and underline in the revised manuscript

Reviewer #3: This is pertinent work. The followings are minor comments

Abstract: There is a need to focus more on the research protocol

Response:The Abstract has been reviewed and updated to aligned to the study protocol and perpectives

Background: Will benefit of drafting a chart with trend of cases, policies, vaccination etc.

Response:We appreciated your comments

Research questions are rather generic

Response:Has been adjusted to be more specific.

Supporting documents do not include questionnaire of informed consent

Response.Both the inform consent and questionnaire has been uploaded

Utilization of the findings can be clearer

Response:Usage of the findings has been modified

Thanks

Bakary Sanneh

Corresponding Author,

Ag.Deputy Director-National Public Health Laboratories

National Public Health Laboratories,

Bertil Herding Highway, Kotu

Ministry of Health

Bertil Herding Highway, Kotu

e-mail: sheikbakary@yahoo.com
