# Supplementary material for: Practice of preventive measures and vaccine hesitance for COVID 19 among households in The Gambia, 2021: Study protocol
Source: PLoS One. 2022 Aug 30;17(8):e0270304. doi: 10.1371/journal.pone.0270304 (PMC9426907; doi:10.1371/journal.pone.0270304)
Supplement: Attachment — Submitted filename: Response letter_COVID KAP 18 04_2022.doc [file pone.0270304.s008.doc]

**Ref: NPHL/Adm/2022/90	18^th^ April 2022**

To

**Editor Plos One**

**Dear Sir/Madam,**

# **Re: (** PONE-D-21-36320 ) **:** Practice of preventive measures and vaccine hesitance for COVID 19 among Households in The Gambia, 2021: Study protocol.

# We are pleased with the insightful review and suggestion to improve the quality of this study protocol. We have attempted to response to all the raised issues and updated the manuscript according. These are as follows:

**Comments to the Author**

Reviewer #2: Topic selections and plan of execution of this research well explained. This study will definitely help to find the KAP regarding COVID-19 among the people. Comments has been well addressed.

Response:The study title has been change as recommended in the revised manuscript.

Reviewer #4: (Corrections below refer to the WORD version clean copy of the covid 19 manuscript _ Revised 2022.docx accessed via click)
Summary of the research and overall impression
The topic of this study protocol is of public health significance at the moment .The investigation into people’s practice of COVID 19 prevention and control measures and factors associated with vaccine hesitance is not only interesting but the results will be useful in the Gambia and other sub-Saharan African settings with similar observed resistance to these best practices. The manuscript is presented in an intelligible fashion but the english could be improved upon to bring it up to standard. There are quite a number of grammatical errors in the Abstract and Background sections. The manuscript would benefit from grammar editing. If the following major revisions are carried out, I strongly recommend it for publication in PLOS ONE.
Major Revision
Title: A preferred title (or a similar one along these lines) would be “Knowledge, Attitude towards COVID19 and its prevention; practice of preventive measures; and COVID 19 vaccine hesitance among Households in the Gambia, 2021: Study protocol.
The problems with the title as written by the authors are:
- “practices of COVID 19”. It’s the preventive measures of Covid 19 that are practiced and not COVID 19.
- Knowledge and attitude towards COVID 19 prevention is not clearly brought out in the title. However, one may assume it is subsumed in just COVID 19.

Response:The study title have been changed as recommended in the revised manuscript.

Abstract: Would benefit from grammar editing.
The following statement should include the other issue being investigated i.e vaccine hesitance “There is urgent need to investigate the knowledge, attitude and practices among the Gambians about preventive practices to control COVID-19”.

Response:The revised study protocol have been proofread by one native English speaker and another volunteer.
Background:
Would benefit from grammar editing.
The following sentences should be rewritten for clarity, their meanings are not clear.
- “It was alluded that some of the study participants were positive for COVID 19 different studies had no signs and symptoms of COVID 19 and thus blurring the fight against such a pandemic[5].”
- “In the post vaccination era of COVID 19 pandemic the pooled prevalence of 15% mortality with found to the associated in hospital admissions [6].
Could the authors provide a reference for this statement from previous studies if available “This is particularly important as the adherence to the control measures by the public has been viewed to be suboptimal”.
The authors state “ There is limited information to explain why people are hesitant to take the COVID 19 vaccinations despite massive community engagement activities done”. While this is true, the authors are advised to cite what little information is available after a thorough search of recent literature”.
The last sentence which states the objectives of the study should be changed to “The objective of this study is to establish the knowledge, attitude towards COVID 19 infection and its prevention; practice of preventive measures; and describe the factors associated with Covid 19 vaccination hesitance in the Gambia”.

Response:The revised study protocol have been proofread by one native English speaker and another volunteer.However,have no data published to substantiate the limited data for low uptake for vaccination and practice of COVID preventive measures.Seems to be a grey area and thus essence of this study.
Hypothesis:
Could the hypothesis be rephrased in a more scientific in language

Response:the hypothesis have been revised as recommended.
Research questions:
The authors should add “prevention and control” after “COVID 19” in the three Research questions. Also a fourth research question is needed to cover “the factors associated with Covid 19 vaccination hesitance” mentioned in the objective of the study.

Response:the reaserch question have been revised as recommended.

Sampling Methodology:
The title of Table 2 was same title of Table 1. This must be an error. The correct title would appear to be the sentence above the table i.e “Distribution of selected households for various LGAs”
Data Management, Analysis and Presentation:
The data analysis should include an analysis of factors associated with vaccine hesitance as stated in the objectives: suggested are cross tabulations with independent variables and KAP of COVID 19 prevention

Response:All concerns have been addressed in the revised study protocol.

Thanks

Bakary Sanneh

Corresponding Author,

Ag.Deputy Director-National Public Health Laboratories

National Public Health Laboratories,

Bertil Herding Highway, Kotu

Ministry of Health

Bertil Herding Highway, Kotu

e-mail: sheikbakary@yahoo.com
